# Current and future strategies in radiotherapy for small-cell lung cancer

**Published:** 2020-09-03

**Authors:** N. Rodríguez de Dios, M. Murcia-Mejía

**Affiliations:** ^1^Department of Radiation Oncology, Hospital del Mar, Barcelona, Spain; ^2^Hospital del Mar Medical Research Institute, Barcelona, Spain; ^3^Pompeu Fabra University, Barcelona, Spain; ^4^Department of Radiation Oncology, Hospital Sant Joan Reus, Tarragona

**Keywords:** small-cell lung cancer, lung cancer, radiotherapy, prophylactic cranial irradiation, thoracic radiotherapy, hyperfractionated, hypofractionated

## Abstract

Small-cell lung cancer (SCLC) accounts for 13% of all lung tumors. The standard treatment in patients with limited-stage (LS) disease is thoracic radiotherapy (TRT) combined with chemotherapy. In extensive-stage (ES) SCLC, the importance of consolidation TRT in patients with a good treatment response has become increasingly recognized. In both LS and ES disease, prophylactic cranial irradiation is recommended in patients who respond to treatment. New therapeutic approaches such as immunotherapy are being increasingly incorporated into the treatment of SCLC, although more slowly than in non-small cell lung cancer. Diverse radiation dose and fractionation schemes, administered in varying combinations with these new drugs, are being investigated. In the present article, we review and update the role of radiotherapy in the treatment of SCLC. We also discuss the main clinical trials currently underway to identify future trends.

**Relevance for Patients:**

Radiotherapy is a critical component of multimodality treatment of SCLC. This article can help physicians to improve medical knowledge and find better ways to treat their SCLC patients.

## 1. Introduction

Lung cancer is the most common cancer type and the leading cause of cancer-related death [1]. Small-cell lung cancer (SCLC) is a particularly aggressive histological subtype characterized by a rapid doubling time, a tendency for early dissemination, and in many cases, rapid symptom onset [2]. SCLC comprises about 13-15% of all lung cancer cases [3].

The incidence of SCLC is strongly associated with cigarette smoking. In Western countries, fewer new cases of SCLC are being diagnosed overall as smoking prevalence has decreased; however, in women, the incidence has increased and now accounts for 50% of new cases, this might reflect changes in smoking habits [4,5]. The average age at diagnosis is also rising (typically > age 70), a trend that has implications for the management of this disease [6].

In 2009, the International Association for the Study of Lung Cancer (IASLC) recommended the 8^th^ edition of the Lung Cancer TNM Classification for SCLC staging to define better prognosis and personalized treatment options [7]. However, the Veterans Administration Lung Group stage system from 1957 which defines limited-stage (LS) and extensive-stage (ES) disease is the most common classification used in clinical practice. Patients with disease confined to one hemithorax, with or without the involvement of the mediastinal, contralateral hilar or ipsilateral supraclavicular, or scalene lymph nodes that could safely be encompassed in the radiotherapy field are considered to have LS, whereas those with disease involvement at any other location are considered to have ES [8]. Unfortunately, only 40% of patients are diagnosed with LS; moreover, the proportion of patients diagnosed with ES has increased substantially in recent years due to the inclusion of positron-emission tomography-computed tomography (PET-CT) and magnetic resonance imaging (MRI) in diagnostic protocols [9].

Even with treatment, outcomes are poor, with a median survival of 16-22 months, with a 5-year survival rate <20% in LS and only 8-13 months, and with a 5-year survival rate <2% in ES [10,11]. For this reason, numerous authors have sought to find a role for surgery in well-selected patients with early-stage disease [12]. Similarly, attempts to optimize chemotherapy regimens in recent decades have only marginally improved treatment response rates. For radiotherapy, strategies to improve outcomes have mainly focused on identifying optimal fractionation schedules, treatment volumes, and timing and on reducing treatment-related side effects. Given this context, there is a strong interest in incorporating new technologies and immune therapies into the treatment arsenal. Nonetheless, at present, smoking cessation interventions are still the main approach used to reduce SCLC-related mortality, primarily because such interventions have proven effective in both preventing disease and improving prognosis after diagnosis [13]. The purpose of this article is to present current data and future directions regarding the treatment of SCLC.

## 2. Limited-stage Small-cell Lung Cancer

### 2.1. Stage I – IIA (T1-T2, N0, M0)

Stage I-IIA (T1-T2N0M0) represents <5% of SCLC. The development of the TNM classification system has increased interest in the role of surgery in SCLC. Although only retrospective, non-randomized studies are currently available, several authors suggest that surgical resection should be considered in the initial treatment of stage T1-2N0, based on the reported 5-year survival rate of 57% in stage I patients [14]. Surgical resection yielded a 5-year survival of 44.9% versus 11.3% compared to conventional treatment in patients with peripheral stage IA-IB disease [15]. The preferred operation is lobectomy with a mediastinal lymph node dissection after the standard staging evaluation [16]. All authors emphasize the importance of comprehensive staging using PET-CT and complete mediastinal evaluation before surgery [17]. Adjuvant chemotherapy following tumor resection is also recommended [18-20]. Several authors have explained the rationale for surgery in SCLC, including the coexistence of mixed histologies, the presence of small lung nodules (i.e., typical or atypical carcinoids) misdiagnosed as SCLC, and the fact that salvage surgery may be preferable to second-line chemotherapy in cases with a local recurrence after an initial response to chemotherapy or if a new tumor appears within 2 years of successful treatment [21,22]. Adjuvant mediastinal radiotherapy after surgical resection is recommended in pN1 and pN2 disease, administered sequentially or concomitantly to chemotherapy. Adjuvant mediastinal radiotherapy improved median survival in pN1 and pN2 disease and may also be associated with longer survival in patients undergoing sublobar resection [23].

Selected patients with stage I-IIA who are medically inoperable due to medical comorbidities, functional status, poor lung function, or refusing surgery may be candidates for stereotactic body radiation therapy (SBRT) [24]. One multicenter study has reported their experience with SBRT in patients with stage T1-2N0 SCLC. All patients were prescribed 50 Gy in five fractions describing a local control of 97.4% at 1 year and 96.1% at 3 years, 1- and 3-year median disease-free survival (DFS), respectively, of 58.3% and 53.2%. Median overall survival (OS) was 17.8 months (69.9% at 1 year and 34% at 3 years). These results were achieved with low toxicity (only 5.2% developed grade ≥2 pneumonitis), leading the authors to conclude that SBRT should be considered as a treatment option for early-stage SCLC [25]. Sequential chemotherapy after SBRT demonstrated an OS of 31.4 versus 14.3 months compared to SBRT alone [26].

### 2.2. Stage IIB - IIIC (T3-4, N0, M0; T1-4, N1-3, M0)

Concurrent chemotherapy and thoracic radiotherapy (TRT) are the standard treatment established. The recommended chemotherapy regimen is platinum-etoposide (PE), maximum of 4-6 cycles, which has been proven to increase survival with less toxicity than other chemotherapy regimens that combine anthracyclines, vinca alkaloids, methotrexate, and/or cyclophosphamide [27,28].

Results from two meta-analysis showed that the use of TRT associated to chemotherapy compared to chemotherapy alone improves intrathoracic disease control at 2 years (47% vs. 24%, respectively) and OS at 3 years by 5.4% consolidate the role of TRT in the LS [29,30].

However, the optimal dose and fraction schedule remains controversial. Two different groups compared the effectiveness of alternating to sequential TRT-chemotherapy and concurrent to alternating TRT-chemotherapy. However, none of the studies found significant differences between these treatment schedules [31,32]. A phase III study compared concurrent to sequential TRT-chemotherapy. In that study, hyperfractionated TRT 45 Gy (1.5 Gy twice daily) was delivered either on the second day of the first cycle or after four sequential cycles, with PE chemotherapy administered in both treatment arms. The results showed a non-significant trend toward improved OS in the concurrent treatment group (27 vs. 20 months, *P*<0.10) [33]. Recently, a preliminary data of a randomized phase II trial comparing high-dose TRT of 60 Gy in 40 fractions (twice daily in 4 weeks) with the standard 45 Gy in 30 fractions (twice daily for 3 weeks) were reported, showing a significant improvement in OS (42 months vs. 23 months). There were no significant differences about toxicity [34].

On the other side, data from accelerated hypofractionated thoracic radiation therapy (HypoTRT) has been reported in the literature, 40 Gy in 16 fractions (once daily) with concurrent chemotherapy followed by prophylactic cranial irradiation (PCI), it appears to be well tolerated [35]. A phase II study showed that HypoTRT of 55 Gy at 2.5 Gy per fraction daily combined with concurrent chemotherapy in LS has favorable survival and acceptable toxicity [36]. A randomized phase II trial comparing hyperfractionated 45 Gy in 30 fractions (twice daily) with HypoTRT 42 Gy in 15 fractions (once daily) in LS reported a higher rate of complete responses (CR) and the longer median OS in hyperfractionated schedule but not statistically significant. There was no difference in toxicity between the two TRT schedules [37].

Regarding timing, early irradiation could reduce the population of chemoresistant cells that may be responsible for treatment failure. Early irradiation could also minimize cellular repopulation. However, delaying TRT after chemotherapy has been completed also has advantages, as the residual tumor volume is likely to be smaller and consequently, the healthy tissue would also be spared, thereby reducing side effects. Although a significant benefit for early TRT has not been observed in all studies, a systematic review showed a small but significant improvement in 2-year OS (5.2%) in patients who received early TRT within the first 9 weeks from the start of chemotherapy [38]. A subgroup analysis showed that this approach was most beneficial in patients who received hyperfractionated TRT and platinum-based chemotherapy.

The time elapsed from the start to end of radiotherapy (SER) is an important predictor of outcome and significantly associated with OS. The 5-year OS with early TRT and SER <30 days was > 20% [39].

A meta-analysis compared earlier to later radiotherapy or shorter versus longer radiotherapy. Early radiotherapy was defined as radiotherapy initiated within 9 weeks from the start of chemotherapy but before the third cycle. Short versus longer course radiotherapy was defined as a difference of ≥2 weeks in treatment duration. OS was significantly better in early or shorter radiotherapy when all chemotherapy cycles were completed (hazard ratio 0.79) [40].

Target volumes definition should include the involved mediastinal nodes and the primary tumor. Two-phase II studies evaluated patients in which only the primary tumor and the positive nodal areas on the pre-treatment computed tomography scan were irradiated. Those studies found isolated nodal recurrence in 5% and 11% of patients, respectively [41,42]. PET-CT not only improves staging but also treatment planning. In one study, only the positive lymph nodes on pre-treatment PET-CT were irradiated, resulting in a low rate (3%) of isolated nodal recurrence [43].

The optimal dose of TRT has not been definitively established. The Intergroup 0096 trial is considered a landmark in the treatment of SCLC. Patients were randomized to either 45 Gy in 5 weeks (25 daily sessions of 1.8 Gy) or 45 Gy in 3 weeks (1.5 Gy, twice daily, 30 fractions) beginning with the first of four PE cycles. The 5-year OS in the hyperfractionated group was 26% versus 16% in the conventional treatment group (*P*=0.04) and local failure was also lower in the hyperfractionated group (36% vs. 52%, *P*=0.06), although acute toxicity (Grade 3 esophagitis) was higher (27% vs. 11%; *P*<0.001) [44]. Although twice-daily radiotherapy concurrently with chemotherapy was established as a standard of care, concern about toxicity, logistical issues, and low dose in the control group resulted in the poor adoption of this regimen [45,46].

The CONVERT trial is a phase III designed to compare the standard 45 Gy in 30 fractions (twice daily for 3 weeks) to 66 Gy in 33 fractions (once daily over 6.5 weeks) starting on day 22 of the chemotherapy cycle 1 (4-6 cycles of cisplatin 25 mg/m^2^ on days 1-3 or 75 mg/m^2^ on day 1 with etoposide 100 mg/m^2^ on days 1-3), followed by PCI if indicated. At a median follow-up of 45 months, no significant between-group differences were found in 1-, 2-, or 3-year OS (respectively: 83% vs. 76%; 56% vs. 51%; and 43% vs. 39%). The acute toxicity profile is comparable except for higher rates of G3-G4 neutropenia in the hyperfractionated group, without any differences in chronic toxicity. This trial was designed to show the superiority of one daily radiotherapy and was not powered to show equivalence, twice daily radiotherapy should continue to be the standard of care in this group of patients [47]. The CONVERT trial reports a lower toxicity due use of PET-CT, 3D conformal radiotherapy or IMRT techniques, and no elective nodal radiation [48].

The CALGB 30610/RTOG 0538 is a phase III study started as a 3-arms study designed to discontinue one of 2 experimental TRT arms based on interim toxicity assessment. Both TRT regimens, 70 Gy daily and 61.2 Gy concomitant boost, concurrent with cisplatin and etoposide chemotherapy, appear to be tolerable without unexpected toxicity. Results of comparing 70 Gy daily in 7 weeks against standard 45 Gy in 30 fractions (twice daily) are near completion [49].

### 2.3. Prophylactic cranial irradiation

The standard chemotherapy for SCLC has a poor effect over the central nervous system that is associated with developing brain metastases (BM). PCI in patients with LS demonstrated a decrease in BM incidence at 3 years (59% vs. 33%) with an absolute advantage of 5.4% in OS [50].

A randomized clinical trial evaluated the dose-response effect of PCI in patients with LS in CR. No significant difference in the incidence of BM was observed between the higher-dose 36 Gy in 18 fractions at 2 Gy daily or 24 fractions at 1.5 Gy, two daily sessions vs. standard-dose 25 Gy in 10 fractions at 2.5 Gy daily. However, 2-year OS was significantly worse in the high-dose group (37% vs. 42%) [51]. Other studies suggest that higher doses are more toxic. Advanced age and high doses >25 Gy show a significant increase in the incidence of chronic neurotoxicity [52].

PCI is associated with improved survival in early-stage patients who undergo complete surgical resection as the initial treatment. A subgroup analysis of the retrospective study showed a significant improvement in stage II-III patients but not in stage I patients, possibly due to the low incidence of BM in that group [53].

A review evaluated PCI in surgically treated stage T1-2N0 patients who underwent rigorous preoperative staging, showing that BM and survival did not differ significantly whether patients receive PCI or not; in addition, PCI-related neurological toxicity was found to be minimal. The authors conclude that PCI could be omitted in surgically resected stage T1-2N0 patients provided that close follow-up with MRI is performed [54]. Overall, PCI improved progression-free survival (PFS) and reduced early incidence of BM, retrospective studies suggest that patients with LS and CR, PCI did not relate the survival benefit when MRI and stereotactic radiosurgery (SRS) were available [55,56].

In summary, stage I-IIA may be treated with surgery or SBRT followed by adjuvant chemotherapy and the standard approach to LS is concurrent PE chemotherapy and TRT administered with the 1^st^ or 2^nd^ cycle followed by PCI for patients who respond to treatment. Hyperfractionation 45 Gy in twice-daily 1.5 Gy fraction remains the standard approach to radiotherapy, but 66-70 Gy in 2 Gy- daily fractions or 60 Gy in twice-daily 1.5 Gy fraction can be considered.

## 3. Extensive-stage Small Cell Lung Cancer

Classically, the standard treatment for ES-SCLC is 4-6 cycles of PE chemotherapy, which achieves a median OS ranging from 8 to 13 months. However, even with objective response rates of 60-80%, only 15-20% of patients achieve CR and prognosis is poor (5-year survival is only 1%) due to recurrence, mainly in the thorax or brain [11,57]. The first-line treatment of ES-SCLC has changed recently with the use of the combination of chemotherapy and immunotherapy. This combination is currently considered the standard first-line treatment of ES-SCLC. The randomized phase III Impower 133 trial showed a significant benefit in OS when atezolizumab, a PD-L1 inhibitor, was added to first-line chemotherapy (carboplatin and etoposide) and continued into the maintenance phase [58]. The phase III CASPIAN trial evaluated durvalumab, with or without tremelimumab plus chemotherapy (etoposide and cis/carboplatin). The planned interim analysis of OS for durvalumab demonstrated a statistically significant improvement in OS [59]. One of the differences between the CASPIAN trial and the IMpower133 trial, which was interesting, was that the CASPIAN trial allowed patients with asymptomatic untreated BM to go on trial without prior brain radiation. Finally, pembrolizumab has been evaluated in patients with newly diagnosed ES-SCLC in combination with chemotherapy (cisplatin/carboplatin plus etoposide) in phase III, randomized, double-blind, and placebo-controlled trial (KEYNOTE 604) [60]. These data show a clear benefit with the addition of PD-L1 therapy to front line chemotherapy with 2-year OS rates of 22%.

### 3.1. Prophylactic cranial irradiation

Approximately 50% of patients with SCLC develop BM. In the meta-analysis by Aupérin *et al*. [50], 15% of patients had ES and PCI was equally beneficial in both ES and LS.

In 2007, a phase III EORTC RCT [61] allocated 286 patients (any response to chemotherapy) to PCI or observation, finding that PCI significantly reduced the risk of symptomatic BM (40% vs. 15% at 1 year) and increased DFS and OS (27.1% vs. 13.3%), without a significant impact on the quality of life. A follow-up study [62] reported details about toxicity, alopecia, and fatigue, all of which were significantly higher in patients who underwent PCI; importantly, however, PCI was well-tolerated, and these side effects did not negatively influence patient self-assessment about their overall health status. The authors conclude that physicians should be alert to possible toxicity, inform patients about the risk, and provide clinical and psychosocial support during follow-up.

Similarly, meta-analysis [50,63-65] and some pooled analysis [66,67] confirmed the EORTC findings described above. Schild *et al*. [68] reported better OS at 1 and 3 years (56% and 18%) in patients treated with PCI versus only 32% and 5% in patients without PCI (HR 0.61, *P*<0.0001). After adjusting for age, performance status, stage, CR, and the number of metastatic locations, hazard ratio remained significant. However, PCI was associated with more grade 3 side effects, especially alopecia and lethargy. Survival was better in patients treated with 25 Gy in 10 fractions. A sub-analysis of the NCCTG trial that included only elderly patients (≥70 years) found that the survival benefit was maintained even in these older patients [61].

Thus, PCI became the standard of care in the ES-SCLC setting; however, a meta-analysis questioned the OS benefit of PCI in ES-SLC [69]. A Japanese randomized trial recently showed that PCI did not increase OS for patients with ES-SCLC. At a planned interim analysis in 2013, the study was terminated early due to futility. Median OS was 11.6 and 13.7 months for the PCI and observation groups, respectively. The incidence of BM in the PCI group was significantly lower, but anorexia and discomfort were slightly more frequent. It should be noted that no quality of life and limited neurocognition data are available from this trial [70].

The divergence between the EORTC results and those of Takahashi *et al*. could be explained by the use of MRI, which detects asymptomatic BM in 25-35% of patients with a CR. Slotman *et al*. did not use MRI before PCI nor in the follow-up of the control group, except when symptomatology suggestive of BM was observed. By contrast, Takahashi *et al*. performed MRI on all patients. As the authors report, physicians in countries other than Japan should be cautious when extrapolating the results of this study to general practice in view of different ethnicities and medical situations. A new analysis performed by Slotman *et al.*, excluding trial patients who had died or developed BM in the first 8 weeks after randomization, confirmed the beneficial effect of PCI [71].

Taken together, these data demonstrate evidence that PCI is associated with a significant reduction in the incidence of BM in ES-SCLC. However, the benefit of PCI on OS may be debatable if patients receive routine surveillance imaging with an MRI brain. Moreover, the majority of patients who do not receive PCI will require salvage whole-brain RT (WBRT) for the treatment of BM.

Consensus analysis of a group of 13 European experts from the European Society for Therapeutic Radiation Oncology and IASLC was performed. It showed that European radiation oncologists and medical oncologists specializing in lung cancer recommend PCI in selected patients and restrict its use primarily to fit, non-elderly patients who responded to chemotherapy [72].

### 3.2. Thoracic radiotherapy

After chemotherapy, 75% of patients present persistent intrathoracic disease and 90% develop intrathoracic progression in the 1^st^ year [61]. Improved staging with PET-CT has resulted in an increase in patients diagnosed with ES versus LS. The meta-analyses by Pignon *et al*. [29] and Warde *et al*. [30] confirmed the benefits of TRT in many patients who today would be diagnosed with ES-SCLC. The role of TRT was addressed in tree randomized controlled trial [73-75] and one meta-analysis [76].

Jeremic *et al*. [73] conducted a randomized controlled trial to assess the association between intrathoracic tumor control and OS. The study included 206 patients <70 years of age with a Karnofsky index ≥70%. After three cycles of PE, patients with a local and distant complete response (CR/CR) or local partial response and distant complete response (PR/CR) (109 patients, groups 1 and 2) were randomized to 2 more cycles of PE or hyperfractionated TRT concomitant with a daily low dose of PE chemotherapy. PCI was later administered in both groups. Patients who experienced less response to chemotherapy were not randomized (groups 3, 4, and 5). OS was higher in the TRT arm (17 vs. 11 months; 5-year OS: 9.1% vs. 3.7%, respectively; *P*=0.041), although the incidence of grade 3 esophagitis was also greater. The best results were observed in the groups (CR/CR or PR/CR) with the best prognostic features after induction chemotherapy. However, these results have not been extended to clinical practice.

In 2015, Slotman *et al*. [74]. reported the results of a multicenter phase III trial, called CREST, in which patients with ES-SCLC were randomized to receive either consolidative TRT or no TRT. In this trial, 495 patients with ES-SCLC with any response after four to six cycles of PE chemotherapy were randomized to receive 30 Gy of TRT in ten fractions or no TRT within 6 weeks of chemotherapy completion. All patients in the study received PCI. While the primary endpoint, OS at 1 year, did not show a statistically significant difference between the two groups, a secondary planned analysis of OS at 2 years was performed. This showed a 2-year OS of 13% (95% CI 9-19) in the TRT group versus 3% (95% CI 2-8; *P*=004) in the no TRT group. Similarly, the secondary endpoint of progression-free survival was better in the TRT group (24%, 95% CI 19-30) than no TRT group (7%, 95% CI 4-11; *P*=0.001). Furthermore, intrathoracic progression was significantly lower, occurring in 43.7% of patients with TRT versus 79.8% of patients with no TRT (*P*<0.001). These results also held true for intrathoracic progression as the first site of relapse (41.7% vs. 77.8%, *P*<0.001) and as the only site of relapse (19.8% vs. 46.0%, *P*<0.001). Severe toxicity was low, with only 1.6% of patients experiencing grade 3 acute esophagitis and 4.5% grade 3 fatigue. The authors conclude that TRT plus PCI should be considered in all patients with ES-SCLC who respond to chemotherapy. A *post hoc* analysis showed that OS was higher in patients with post-chemotherapy residual chest disease and no benefit was seen for thoracic CR (HR 0.81; 95% CI 0.66-1.00, *P*=0.044). Another secondary analysis of the study concluded that the OS (p = 0.02) and PFS (*P*=0.04) were significantly better in patients with 2 or fewer metastases, with OS significantly worse if liver (*P*=0.03) and/or bone metastases (*P*=0.04) were present [77].

However, the high rates of initial intrathoracic progression in this study may be atypical in the general ES-SCLC population, where the widespread metastatic disease is often present upon recurrence. More than 40% of patients who received TRT had an intrathoracic recurrence, which might suggest that even higher doses of radiation might be more efficacious. In a prospective non-randomized phase 2 study of patients with ES-SCLC given four cycles of platinum-based chemotherapy, subsequent consolidative TRT delivered as 40 Gy in 15 daily fractions was well tolerated, and only five of 32 patients developed a symptomatic chest recurrence [78].

The RTOG 0937 trial included patients with ES disease with 1-4 sites extracranial metastatic disease who achieved a PR/CR to systemic therapy. Patients with BM were excluded from the study. Patients were randomized to receive PCI with or without radiotherapy to the thorax as well as sites of distant metastatic disease. In contrast to the CREST study, patients were recommended to receive a dose of 45 Gy in 3 Gy fractions to the thorax and 30-45 Gy to sites of distant disease. The primary endpoint of the study was OS and planned accrual was 154 patients. However, the study accrued very slowly and was terminated early after 97 evaluable patients were enrolled to the study due to a pre-planned interim analysis that showed the study crossed the futility boundary for OS. One-year OS was better than expected in both arms: 60.1% in the PCI arm and 50.8% in the PCI + TRT arm (compared to 27% in the best arm of EORTC PCI study). Time to progression showed a significant benefit for the patients who received local radiotherapy. The pattern of failure analysis demonstrated that consolidative radiotherapy to all sites of extracranial disease following PCI reduced the risk of the first failure in the thorax from 62.5% to 25.8% and reduced the risk of failure at one of the sites of presenting metastatic disease from 78.1% to 41.9% [75].

The meta-analysis by Palma *et al*. [76] concluded that TRT increases OS and PFS in patients with ES-SCLC who respond to the initial chemotherapy, with only a small increase in the risk of esophageal toxicity.

To summarize the role of radiotherapy in ES-SCLC, we can state that the administration of PCI in chemotherapy responders decreases the incidence of symptomatic BM and may increase OS. The standard dose is 25 Gy in 10 daily fractions. If PCI is not prescribed, MRI-based brain imaging should be performed for early detection of BM given the potential benefit of SRS in these cases.

TRT improves OS and should be considered in ES-SCLC patients whit no progression after chemotherapy (especially for residual thoracic tumor). A minimum dose of 30 Gy in 10 fractions is recommended.

## 4. Future Directions

The availability of increasingly accurate imaging systems in modern treatment units allows us to modify the treatment plan according to temporary changes in anatomy (tumor reduction, atelectasis, weight loss, internal movement, etc.) as well as according to changes in the biology/function of the tumor (proliferation, hypoxia, etc.). This ability to modify the treatment is known as adaptive radiotherapy, an approach that is especially valuable in SCLC because this tumor type is more sensitive than other types to chemotherapy and radiotherapy. Yee *et al*. [79] performed serial 4D-CT scans to quantify changes in tumor volume during concomitant chemoradiotherapy, finding that the maximal reduction in volume occurred early (i.e., after the 1^st^ week) in the treatment process. The ability to adapt the treatment plan to match the reduced tumor volume would allow us to simultaneously increase the dose to the tumor while reducing the dose to organs at risk, which would also decrease toxicity [80]. This approach has also been shown to improve local control [81]. Recent advances in medical imaging technology allow the use of more advanced image analysis methods beyond simple measurements of tumor size or radiotracer uptake metrics. Radiomics is an emerging field of quantitative imaging that aims to extract quantitative data from medical images to characterize tumor histology or heterogeneity using a large set of advanced imaging features. Radiomics has several implications in lung cancer. Several papers have shown that the combination of clinical, genomic, and radiomic features provides information that may be useful to guide the therapies and predict survival. Ramella *et al*. [82] investigated the feasibility of a system where the radiomic features of the non-small cell lung cancer (NSCLC) patient’s initial imaging were able to predict tumor reduction during chemoradiation. Recently, also prediction using radiomic analyses of cone-beam CT images has been reported [83].

There are several unanswered questions regarding the treatment of ES-SCLC. For example, we do not know the optimal radiotherapy dose for thoracic consolidation, or the indication for this technique in patients with thoracic CR or distant PR. A phase III trial (Clinical Trials. Gov. Identifier: NCT02675088) is currently being conducted to compare 45 Gy at 3 Gy/day in 15 fractions versus 30 Gy in 10 fractions (the dose used in the CREST trial) [70] with the primary endpoint being 2-year OS. In terms of the design of future studies, the use of PET-CT scans would be valuable to better assess the primary tumor and the location of the metastatic lesions – such information would help to improve patient selection. In addition, given recent technological advancements, it is now possible to deliver radical doses to treat more than one location simultaneously; hence, detailed data on the metastatic lesions are crucial. Additional research on TRT and immunotherapy is needed to address indications, timing and dose to combine both treatments in ES-SCLC patients.

Following on from positive trials adding immunotherapy to the standard of care treatment in ES-SCLC an ongoing question is whether immunotherapy could improve survival in the LS setting. There are three randomized trials currently investigating the addition of immunotherapy to chemoradiotherapy in LS-SCLC. NRG Oncology and Alliance are currently testing the use of atezolizumab after chemoradiotherapy in the LS-SCLC setting (Clinical Trials. Gov. Identifier: NCT03811002). Another trial utilizing immunotherapy for the treatment of LS-SCLC is the European Thoracic Oncology Platform (ETOP) sponsored STIMULI trial which is a phase II randomized trial investigating if the use of consolidation with nivolumab and ipilimumab in LS-SCLC after chemoradiation therapy and PCI is better than chemoradiation therapy and PCI alone (Clinical Trials. Gov. Identifier: NCT02046733); another clinical trial testing the role of immunotherapy is the phase III, randomized, double-blind, international study ADRIATIC study sponsored by AstraZeneca (ClinicalTrials.gov Identifier: NCT03703297). This is a three-arm study evaluating the efficacy of durvalumab or durvalumab with tremelimumab compared to placebo for consolidation in LS-SCLC patients who have not progressed after concurrent chemoradiation.

The benefit of PCI was called into question with the publication of a Japanese randomized trial. Contemporary use of MRI and head computed tomography staging detects BM in approximately one-quarter of patients with SCLC at diagnosis and in an additional one-third of patients after initial therapy [84]. A recent meta-analysis of 7 similar randomized trials, which included more than 2000 patients, found substantial heterogeneity in their OS analysis, which the authors thought was associated primarily with the heterogeneity in imaging protocols among the various trials [85]. Pezzi *et al*. [86] retrospectively compared the rates of intracranial control and OS data in patients with LS-SCLC, all of whom underwent staging with MRI, who were treated with or without PCI. The 3-year cumulative incidence rate of BM was higher in the no-PCI group versus the PCI group when counting death as a competing risk, but the difference was not statistically significant (20.40% [95% CI, 12.45%-29.67%] vs. 11.20% [95% CI, 5.40%-19.20%]; *P*=0.10). The use of PCI was not associated with a difference in OS between the patient groups (hazard ratio, 0.844; 95% CI, 0.604-1.180; *P*=0.32). There is one ongoing phase III trial (S1827/MAVERICK trial, Clinical Trials. Gov. Identifier: NCT04155034) evaluating the role of PCI versus MRI surveillance in patients with both LS and ES SCLC. A similar study is in set up within the EORTC as well.

In recent years, researchers have sought to reduce PCI-related toxicity, especially the deleterious effects on neurocognitive function. Several factors have been associated with increased cognitive impairment in patients treated with PCI, including the administration of daily doses > 3 Gy, higher total doses, the use of chemotherapy, and patient-related factors (age, chronic tobacco exposure, diabetes, and depression, among others) or to the disease itself (undiagnosed micrometastases, paraneoplastic syndromes, etc.). Both preclinical and clinical studies have shown that one of the most important neurocognitive changes associated with WBRT is a selective negative effect on memory due to a decrease in hippocampal neurogenesis [87], with a dose-response relationship between the radiation dose to the hippocampus and the risk of memory and learning impairment [88]. Given the very low (5%) risk of hippocampal metastasis [89], together with the promising results achieved in patients whose BM were treated with hippocampal-avoidance WBRT [90], three-phase III studies (NKI/AVL Clinical Trials. Gov. Identifier: NCT01780675; NRG CC003 Clinical Trials. Gov. Identifier: NCT02635009 and PREMER-TRIAL Clinical Trials. Gov. Identifier: NCT02397733 [91]) are underway to evaluate this approach in patients with ES- or LS-SCLC without BM. All three studies share a similar design and the same primary objective: To evaluate the efficacy of hippocampal-avoidance PCI (HA-PCI) to prevent neurocognitive alterations. Two of these studies have been reported in an abstract form. NKI-AVL, where HA-PCI compared to conventional PCI revealed a non-significant decline by ≥5 points at Hopkins Verbal Test Learning-Revised (HVLT-R) total recall score in 28% of the total group [92]. In contrast, PREMER-trial reported a significant decline by ≥3 points at Free and Cued selective Remanding test (FCSRT) delayed free recall 22.22% versus 5.08% (OR 5.33; 95 CI: 1.44-19.65; *P*=0.006) and FCSRT total recall 20.63% versus 6.78% (OR 3.57 [1.09-11.68] *P=*0.02) in PCI versus HA-PCI group [93]. Another related line of research could be the use of Alzheimer’s drugs to reduce the negative effects of radiation-induced toxicity on neurocognitive function due to the excellent results reported in patients with BM [94,95].

Although SRS alone for limited BM has been accepted across most histologies, SCLC represents a notable exception where WBRT remains a guideline recommendation in cases ranging from diffuse to solitary brain lesions. SRS has been relegated to use mainly after failed WBRT, probably due to a lack of evidence of the efficacy of SRS for this malignancy. However, recent refinements in diagnostic and therapeutic modalities, including the expanded use of surveillance brain MRI, evolving controversies surrounding PCI, and integration of immunotherapy into treatment, may impact the modern management of BM. Historic objections to the use of SRS in SCLC have included the concern for diffuse interval SNC progression and the potential for a resulting decrease in survival in such cases. There is, however, growing evidence to suggest that SRS alone may be appropriate for some patients with SCLC [96-98]. One recent phase II trial (ENCEPHALON. Clinical Trials. Gov. Identifier: NCT03297788) is comparing SRS to WBRT for SCLC patients with 1-10 BM.

Proton beam therapy (PBT) represents one of the most advanced radiation modalities for oncologic management. Emerging evidence from NSCLC has demonstrated a significant correlation between radiation dose to the heart and OS, even after correcting for other well-established prognostic factors [99]. This has led to an interest in evaluating the role of PBT as a method to reduce radiation dose to the heart while maintaining the definitive radiation dose required for lung cancer treatment. Although this relationship is best established in NSCLC, it is plausible that a similar phenomenon is seen in SCLC patients, where patients are even more likely to have a bulky central disease that will result in higher radiation doses to the heart using photon radiotherapy.

The results of a prospective study evaluating outcomes for LS-SCLC patients tread with PBT were recently published. This single-institution study from the University of Pennsylvania included 30 patients treated to a median dose of 63.9 Gy cobalt Gy equivalents. The median OS for the cohort was 28.2 months and there few high-grade treatment-related adverse events [100]. Although the results of this prospective study are encouraging, they require validation in future clinical trials.

## 5. Conclusion

The most recent studies of SCLC underscore the importance of using a multimodal approach that includes both chemotherapy and radiotherapy to treat both limited and extensive-stage SCLC (Table 1). At present, the standard radiotherapeutic approach to LS-SCLC is 45 Gy delivered in a continuous hyperfractionated regimen; however, 66-70 Gy in daily fractionation can also be used pending the results of ongoing trials. In ES-SCLC, consolidation TRT with a minimum dose of 30 Gy in 10 fractions should be offered to patients who respond well to chemotherapy. Finally, PCI techniques with hippocampal avoidance are being examined along with the role of MRI surveillance. The results of ongoing large randomized trials along with future research and novel immunotherapeutic and new combinations are expected to define the final role of immunotherapy in the treatment algorithm for SCLC

**Table 1:** Recommendations for radiotherapy in SCLC.

Limited stage	**I-IIA: Surgery** ^[14-16]^
	• SBRT if surgery is contraindicated or the patient refuses surgery^[24,25]^
	• Consider adjuvant systemic therapy^[18-20]^
	• No clear benefit of PCI^[53,54]^
	**IIB-IIIC:**
	• QT RT Concomitant^[29-33]^: Early (<9 weeks or SER <30 days)^[38-40]^ 45 Gy BID^[44]^
	*Alternative*:
	66 Gy OD (CONVERT)^[48]^
	60 Gy BID (Norway)^[34]^
	• Postoperative RT: after surgical resection in pN1 and pN2 disease^[23]^
	**• PCI:**
	25 Gy in 10 fractions^[50-52]^ Consider hippocampal avoidance^[ 92,93]^
Extensive stage	**• Consolidative Thoracic radiotherapy^[73-76]:^**
	Most benefit if there are post-chemotherapy residual chest disease and ≤2 metastases^[74,77]^ 30 Gy in 10 fractions (CREST)^[74]^
	Consider 40-45 Gy in 15 fractions^[75,78]^
	**• PCI**
	25 Gy in 10 fractions^[50,61-67]^ Consider hippocampal avoidance^[92,93]^ Consider MRI surveillance in elderly patients and /or patients with neurocognitive impairment^[70]^

SBRT: Stereotactic body radiation therapy; PCI: Prophylactic cranial irradiation;

SER: Time between the first day of chemotherapy and the last day of chest radiation;

MRI: Magnetic resonance imaging

### Conflicts of Interest

The authors indicate no potential conflicts of interest.
